# LRP5 in age-related changes in vascular and alveolar morphogenesis in the lung

**DOI:** 10.18632/aging.101722

**Published:** 2019-01-05

**Authors:** Akiko Mammoto, Megan Muyleart, Tadanori Mammoto

**Affiliations:** 1 Department of Pediatrics, Medical College of Wisconsin, Milwaukee, WI 53226 USA; 2 Department of Radiology, Medical College of Wisconsin, Milwaukee, WI 53226 USA; * Equal contribution

**Keywords:** aging, angiogenesis, lung, LRP5, VEGFR2, Tie2

## Abstract

Aging is associated with impaired angiogenesis and lung alveolar regeneration, which contributes to the increased susceptibility to chronic lung diseases (CLD). We have reported that the Wnt ligand co-receptor, low-density lipoprotein receptor-related protein 5 (LRP5), stimulates angiogenesis and lung alveolar regeneration. However, the role of LRP5 in age-related decline in vascular and alveolar morphogenesis remains unclear. In this report, we have demonstrated that vascular and alveolar structures are disrupted in the 24-month (24M) old mouse lungs. The expression of LRP5 and the major angiogenic factors, VEGFR2 and Tie2, is lower in endothelial cells (ECs) isolated from 24M old mouse lungs compared to those from 2M old mouse lungs. Vascular and alveolar formation is attenuated in the hydrogel implanted on the 24M old mouse lungs, while overexpression of LRP5, which restores angiogenic factor expression, reverses vascular and alveolar morphogenesis in the gel. Compensatory lung growth after unilateral pneumonectomy is inhibited in 24M old mice, which is reversed by overexpression of LRP5. These results suggest that LRP5 mediates age-related inhibition of angiogenesis and alveolar morphogenesis. Modulation of LRP5 may be a novel intervention to rejuvenate regenerative ability in aged lung and will lead to the development of efficient strategies for aging-associated CLD.

## Introduction

The aging population, aged 65 and older, is rapidly growing and is estimated to reach 83.7 million in 2050 (US Census Bureau). Because of chronic inflammation, oxidative stress, abnormal shortening of telomeres, and changes in local microenvironment and stem cell populations, the aging population is at high risk for CLD including chronic obstructive pulmonary diseases (COPD) [1–6]; the number of COPD patients is 4 times higher in the aging population compared to that in younger populations and COPD is the fourth leading cause of the death in aging people (ALA Epidemiology and Statistics Unit). These figures indicate a critical need for the development of more effective ways to treat CLD in aging people. Impairment of lung regeneration and repair is one of the most important factors in COPD progression [7,8]. It has been reported that compensatory lung growth after unilateral pneumonectomy (PNX) is highly induced in the lungs of juvenile people [9,10], while it is significantly diminished in older people [9,11,12]. Thus, rejuvenation of the intrinsic regenerative ability in aged lungs could be a promising strategy for CLD in aging people.

Wnt signaling plays important roles in lung vascular and alveolar development [13–17]. Wnt signaling is suppressed in aging organs including lungs [18] and activated Wnt signaling inhibits cellular senescence [18–20]. Reduced Wnt signaling is associated with aging-associated diseases such as heart disease [21], Alzheimer’s disease [22], osteoporosis [23], diabetes [24], and COPD [1,25,26]. We and other groups have shown that the Wnt ligand co-receptor, low-density lipoprotein receptor-related protein 5 (LRP5) controls various angiogenic pathways (e.g., angiopoietins (Angs)-Tie2 [15,17], VEGF-VEGFR2 [27,28], neuropilin (NRP2) [29]), and stimulates retinal [27,30–32] and lung vascular development in neonatal mice [14–17]. LRP5 also mediates compensatory lung growth after PNX in young adult mice through Ang-Tie2 signaling [33]. Thus, LRP5 signaling may be the key control point for the impairment of vascular and alveolar morphogenesis in the aged lung.

Here we have demonstrated that angiogenesis and alveolar morphogenesis are impaired in aged mouse lungs through suppression of LRP5 signaling. Activation of LRP5 signaling restores the age-related decline in lung vascular and alveolar morphogenesis. Modulation of LRP5 signaling would be an efficient therapeutic strategy for aging-associated lung diseases.

## RESULTS

### Vascular and alveolar structures are disrupted in the aged mouse lung

**Figure 1:**
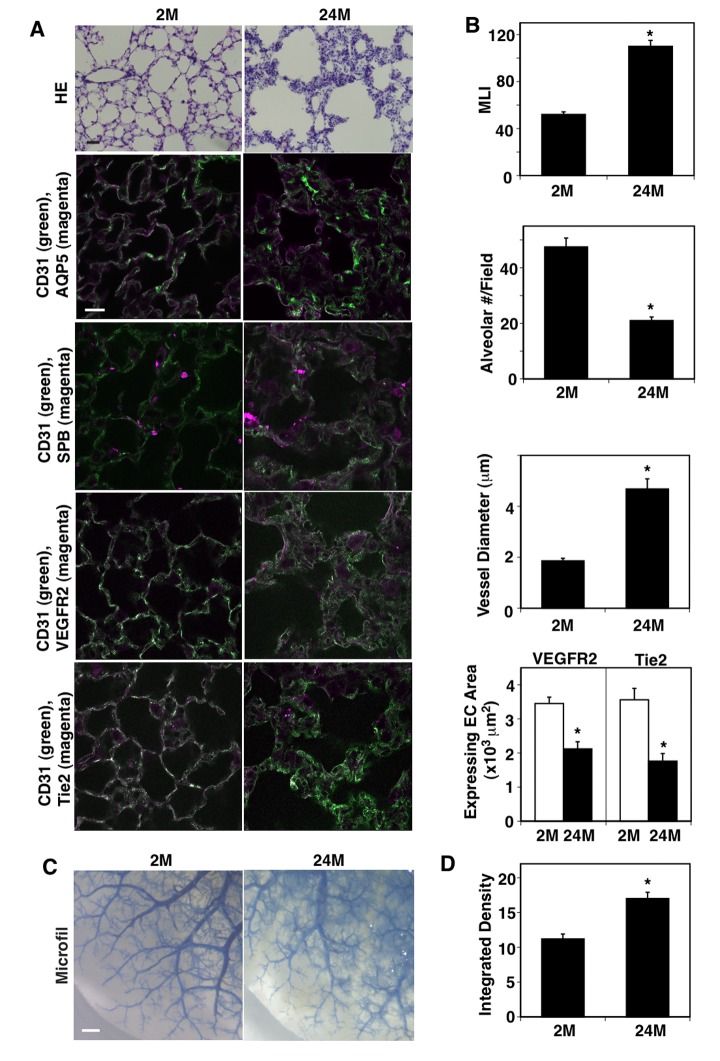
**Age-dependent changes in vascular and alveolar structures in the mouse lungs.** (**A**) H&E-stained 2M and 24M old mouse lungs (*top*, scale bar, 20 μm). Immunofluorescence micrographs showing CD31-positive blood vessels and AQP5-positive alveolar type-I epithelial cells (*2nd*), CD31-positive blood vessels and SPB-positive alveolar type-II epithelial cells (*3rd*), CD31-positive blood vessels and VEGFR2 expression (*4th*), and CD31-positive blood vessels and Tie2 expression (*bottom*) in the 2M vs. 24M old mouse lungs (scale bar, 20 μm). (**B**) Graphs showing quantification of alveolar size (MLI, *top*), alveolar number (*2nd*), vessel diameter (*3rd*), and area of ECs expressing VEGFR2 and Tie2 (*bottom*) in the 2M and 24M old mouse lungs (n=7, mean ± s.e.m., *, p<0.05). (**C**) Micrographs showing blood vessel structures in the 2M and 24M old mouse lungs analyzed using the Microfil casting system. Scale bar, 1 mm. (**D**) Graph showing the quantification of casting reagent leaked out of the lung blood vessels (n=7, mean±s.e.m., *p<0.05).

When we examined vascular and alveolar structures in the young (2 months (2M) old) vs. aged mouse lungs (24M old) using histological (hematoxylin & eosin (H&E) staining) and immunohistochemical (IHC) analyses, the structure of aquaporin 5 (AQP5)- and surfactant protein-B (SPB)-positive alveolar units (septation) and CD31-positive blood vessel structures in the septa were disrupted in 24M old mouse lungs compared to those in 2M old mice (Figure 1A); alveolar size characterized by measuring the mean linear intercept (MLI) was 2.1-times higher, while the alveolar number was 57% lower in the 24M old mouse lungs compared to those in the 2M old mouse lungs (Figure 1B). Although vascular density was not significantly changed in the alveolar septa, vessel diameter was 2.5-times larger and the expression of major angiogenic factor receptors, VEGFR2 and Tie2, in ECs decreased by 39% and 49%, respectively, in the 24M old mouse lungs compared to those in the 2M old mouse lungs (Figure 1B). We also analyzed the age-dependent effects on blood vessel structures using the microfil casting system (Figure 1C). The casting reagents that leaked out of the blood vessels increased by 1.5-fold in the 24M old mouse lungs compared to those in the 2M old mouse lungs (Figure 1C, D).

**Figure 2:**
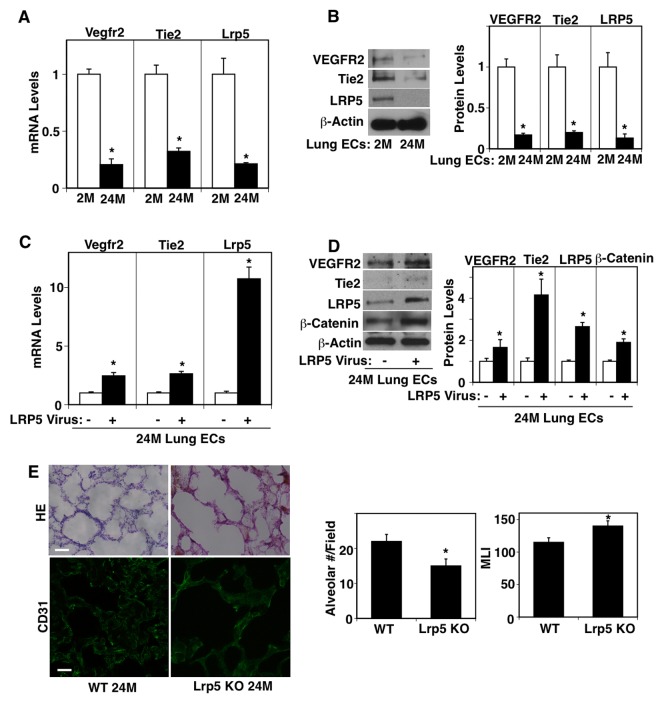
**LRP5 mediates age-dependent changes in angiogenic factor receptor expression in mouse lung ECs.** (**A**) Graph showing the mRNA levels of Vegfr2, Tie2, and Lrp5 in ECs isolated from 2M vs. 24M old mouse lungs (n=4, mean±s.e.m., *, p<0.05). (**B**) Representative immunoblots showing VEGFR2, Tie2, LRP5, and β-actin protein levels in ECs isolated from 2M vs. 24M old mouse lungs. Graph showing VEGFR2, Tie2, and LRP5 protein levels normalized by β-actin protein levels in ECs isolated from 2M vs. 24M old mouse lungs (n=4, mean±s.e.m., *, p<0.05). (**C**) Graph showing the mRNA levels of Vegfr2, Tie2, and Lrp5 in ECs isolated from 24M old mouse lungs treated with lentivirus overexpressing LRP5 (n=4, mean±s.e.m., *, p<0.05). (**D**) Representative immunoblots showing VEGFR2, Tie2, LRP5, β-catenin, and β-actin protein levels in ECs isolated from 24M old mouse lungs treated with lentivirus overexpressing LRP5. Graph showing VEGFR2, Tie2, LRP5, and β-catenin protein levels normalized by β-actin protein levels in ECs isolated from 24M old mouse lungs treated with lentivirus overexpressing LRP5 (n=4, mean±s.e.m., *, p<0.05). (**E**) H&E-stained 24M old WT and Lrp5 KO mouse lungs (*top*, scale bar, 50 μm). Immunofluorescence micrographs showing CD31-positive blood vessels in the 24M old WT and Lrp5 KO mouse lungs (*bottom*, scale bar, 20 μm). Graphs showing quantification of alveolar number (*left*) and alveolar size (MLI, *right*) in the 24M old WT and Lrp5 KO mouse lungs (n=4, mean ± s.e.m., *, p<0.05).

Consistent with the results of aged lung tissues, Vegfr2 and Tie2 mRNA levels were lower by 79% and 68%, respectively, in ECs isolated from 24M old mouse lungs compared to those in the gender-matched 2M old mouse lungs (Figure 2A). The protein levels of VEGFR2 and Tie2 were also lower by 83% and 80%, respectively, in 24M old mouse lung ECs when analyzed using IB (Figure 2B). LRP5 stimulates lung development and regeneration through Ang1-Tie2 signaling [15,17,33] and Wnt signaling and angiogenic signaling are reduced in aging organs including the lungs [18,34–40]. We have reported that Lrp5 knockout (KO) mouse reveals retarded postnatal alveolar development and decreases angiogenic factor expression in the lung [15]. We have also demonstrated that compensatory lung growth after unilateral PNX is inhibited in Lrp5 KO mice [33]. Thus, we next examined the expression of LRP5 in the aged mouse lungs. The mRNA and protein levels of LRP5 were lower by 79% and 86%, respectively in ECs isolated from 24M old mouse lungs compared to those in 2M old mouse lung ECs (Figure 2A, B). Overexpression of LRP5 using lentiviral transduction restored VEGFR2 and Tie2 mRNA and protein expression in ECs isolated from 24M old mouse lungs (Figure 2C, D). LRP5 overexpression also increased β-catenin protein expression in 24M old mouse lung ECs when analyzed using IB and immunocytochemical analysis (Figure 2D, Supplementary Figure S1A), suggesting that LRP5 stimulates angiogenic factor expression in aged ECs through canonical Wnt signaling. When we evaluated the lung vascular and alveolar morphology in 24M old Lrp5 KO mice, vascular and alveolar structures were more severely disrupted in 24M old Lrp5 KO mouse lungs compared to those in 24M old wild-type (WT) mouse lungs (Figure 2E). The alveolar number was 32% lower and MLI was 1.2-fold higher in the 24M old Lrp5 KO mouse lungs compared to those in the 24M old WT mouse lungs. These results suggest that suppression of LRP5 expression mediates age-dependent decreases in VEGFR2 and Tie2 expression in lung ECs and contributes to the inhibition of vascular and alveolar morphogenesis in the aged mouse lung.

### LRP5 mediates age-related decline in angiogenesis and alveolar morphogenesis in the lung

**Figure 3:**
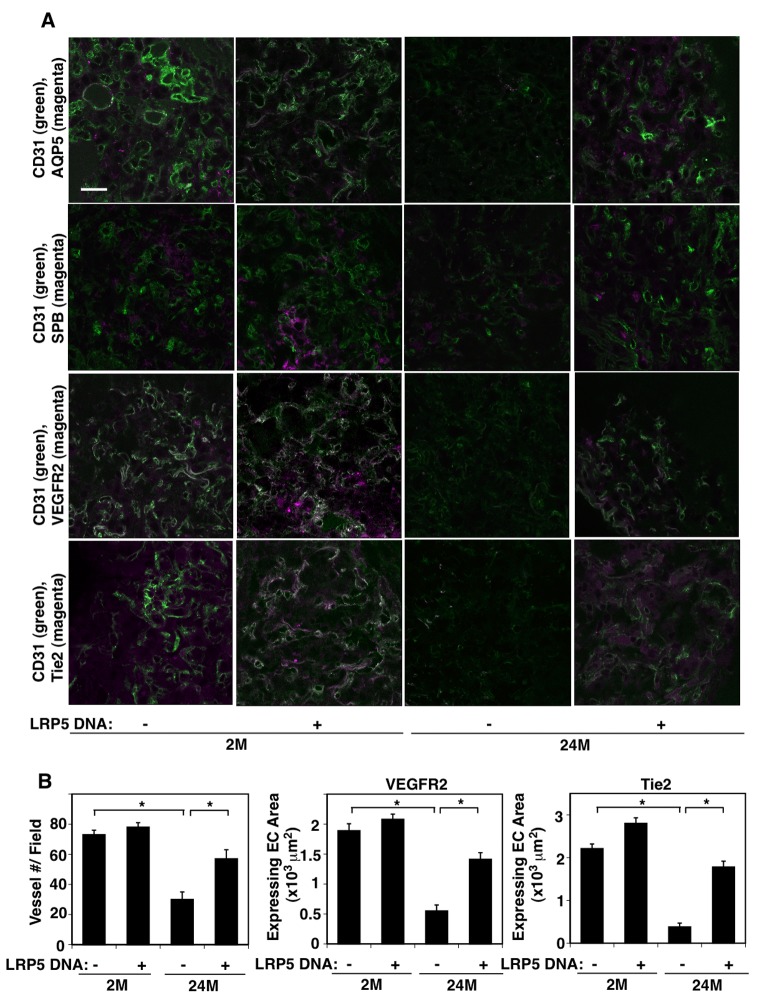
**LRP5 mediates age-dependent decline in vascular and alveolar epithelial morphogenesis in the gel implanted on the mouse lungs. (A)** Immunofluorescence micrographs showing CD31-positive blood vessels and AQP5-positive alveolar type-I epithelial cells (*top*), CD31-positive blood vessels and SPB-positive alveolar type-II epithelial cells (*2nd*), CD31-positive blood vessels and VEGFR2 expression (*3rd*), and CD31-positive blood vessels and Tie2 expression (*bottom*) in the fibrin gel implanted on the 2M vs. 24M old mouse lungs or in combination with LRP5 overexpression for 7 days (scale bar, 20 μm). (**B)** Graphs showing quantification of CD31-positive blood vessel numbers (*left*), area of ECs expressing VEGFR2 (*middle*) and Tie2 (*right*) in the gel implanted on the 2M vs. 24M old mouse lungs or in combination with LRP5 overexpression for 7 days (n=7, mean ± s.e.m., *, p<0.05).

To further study the effects of aging on newly formed vascular and alveolar morphogenesis in the lung, we implanted fibrin gel on the 2M vs. 24M old mouse lungs [41–43] and characterized the vascular and alveolar epithelial morphogenesis in the gel. Consistent with the decreases in the expression of angiogenic factors in aged mouse lung ECs (Figure 2), CD31-positive blood vessel formation, which is well developed in the gel implanted on the 2M old mouse lungs, was attenuated in the gel implanted on the 24M old mouse lungs for 7 days; vascular density was 60% lower than that in the gel implanted on the 2M old mouse lungs (Figure 3A, B). The levels of VEGFR2 and Tie2 were also lower by 70% and 91%, respectively in the gel implanted on the 24M old mouse lungs (Figure 3A, B). AQP5- and SPB-positive alveolar epithelial cells aligned along the ECs in the gel implanted on the 2M old mouse lungs, while these alveolar epithelial morphogenesis was inhibited when the gel was implanted on the 24M old mouse lungs: AQP5- and SPB-positive alveolar epithelial cell area was lower by 61% and 38%, respectively compared to that in the gel implanted on the 2M old mouse lungs (Figure 3A, Supplementary Figure S1B). We also manipulated LRP5 expression in 2M vs. 24M old mice using intravenous injection of LRP5 DNA (retroorbital injection, twice/week) [43]. Consistent with others’ reports [44,45], LRP5 was expressed not only in CD31^+^, VE-cadherin^+^, CD45^-^ EC populations but also in other types of cells, including EpCAM^+^ alveolar epithelial cells and alveolar immune cells (Supplementary Figure S1C). LRP5 DNA intravenous injection increased Lrp5 mRNA levels in CD31^+^, VE-cadherin^+^, CD45^-^ EC populations and immune cells in the BAL fluid by 2.8- and 3.7-times, respectively when analyzed using qRT-PCR (Supplementary Figure S1D). Intravenous injection of LRP5 DNA also reversed angiogenesis, alveolar morphogenesis, and angiogenic factor expression inhibited in the gel implanted on the 24M old mouse lungs (Figure 3, Supplementary Figure S1B). These results suggest that LRP5 mediates age-dependent decline in angiogenesis and alveolar morphogenesis in the gel implanted on the mouse lungs.

### LRP5 mediates age-related inhibition of compensatory lung growth after PNX

**Figure 4:**
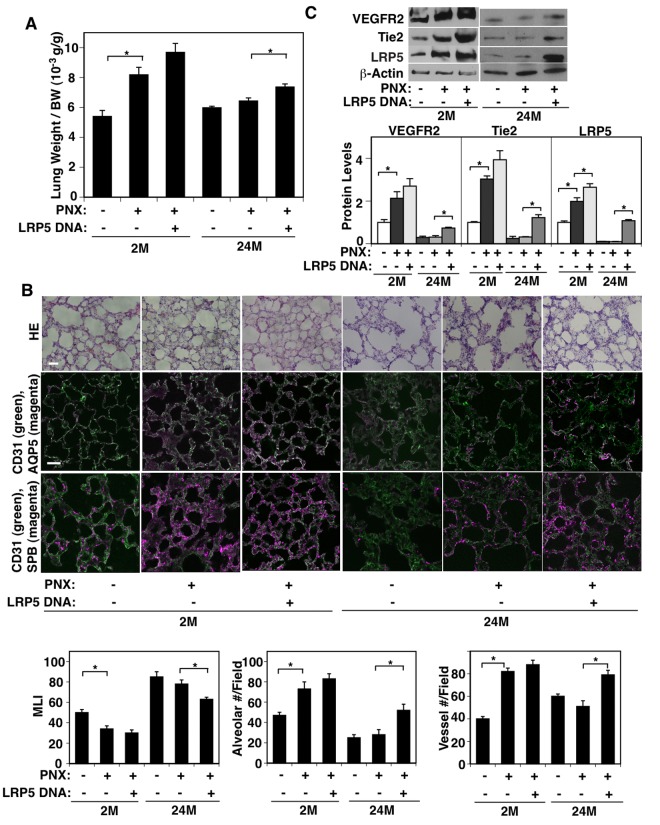
**LRP5 mediates age-dependent inhibition of post-PNX compensatory lung growth.** (**A**) Graph showing the ratio of the weight of right lung cardiac lobe to mouse BW in the 2M vs. 24M old mice after PNX or in combination with LRP5 overexpression for 7 days after PNX (n=7, mean ± s.e.m., *, p<0.05). (**B**) H&E-stained mouse lungs (*top*, scale bar, 20 μm), CD31-positive blood vessels and AQP5-positive alveolar type-I epithelial cells (*middle*, scale bar, 20 μm), and CD31-positive blood vessels and SPB-positive alveolar type-II epithelial cells (*bottom*) in the cardiac lobe of 2M vs. 24M old mice after PNX or in combination with LRP5 overexpression for 7 days after PNX. Graphs showing quantification of alveolar size (MLI, *left*), alveolar number (*middle*), and vessel number (*right*) in the cardiac lobe of 2M vs. 24M old mice after PNX or in combination with LRP5 overexpression for 7 days after PNX (n=7, mean ± s.e.m., *, p<0.05). (**C**) Representative immunoblots showing VEGFR2, Tie2, LRP5, and β-actin protein levels in the 2M vs. 24M old mouse lungs after PNX or in combination with LRP5 overexpression for 7 days after PNX. Graph showing the quantification of immunoblots (n=4, *, mean ± s.e.m., *, p<0.05).

It has been reported that compensatory lung growth after unilateral PNX is significantly diminished in older people [9,11,12]. We have demonstrated that LRP5 mediates compensatory lung growth after PNX in young adult mice [33]. Thus, we next examined whether aging suppresses compensatory lung growth after PNX through LRP5 signaling using a mouse unilateral PNX model. Consistent with previous reports [33,46–48], there was a significant increase in the ratio of the weight of right cardiac lobe to mouse body weight (BW) 7 days after left unilateral PNX on 2M old mice; the lung weight to BW ratio was 5.4 x10^-3^ (g/g) in the sham-operated control mice, while the ratio increased by 1.4-fold in the lungs 7 days after PNX (Figure 4A). Morphometric analysis of H&E-stained mouse lungs also revealed that the size of the alveolar space measured by MLI decreased by 33%. The number of alveoli and CD31-positive blood vessels increased by 1.7- and 2.1- times, respectively, in the remaining lung lobe after left PNX compared with control sham-operated 2M old mouse lungs (Figure 4B). However, these post-PNX effects on compensatory lung growth and vascular and alveolar morphogenesis were attenuated in the 24M old mouse lung after PNX (Figure 4A, B). The protein levels of LRP5 and angiogenic factor receptors, VEGFR2 and Tie2, also increased by 1.9-, 2.1-, and 2.9-times in the 2M old mouse lungs 7 days after left PNX compared to those in the sham-operated control mouse lungs, while these increases were suppressed in 24M old post-PNX mouse lungs (Figure 4C).

To study whether LRP5 mediates age-dependent inhibition of compensatory lung growth after PNX, we treated mice with LRP5 DNA using intravenous injection (retroorbital injection, twice/week) [43] after unilateral PNX. LRP5 DNA injection, which increased the LRP5 protein expression by 5.2-fold in the 24M old mouse lungs 7 days after treatment (Figure 4C), restored the lung growth 7 days after left unilateral PNX in 24M old mice compared to that in the control-vector injected mouse lungs (Figure 4A). LRP5 overexpression also restored the size of the alveolar space measured by MLI, the number of alveoli expressing AQP5 and SPB, and the vessel numbers in 24M old mouse lungs after left unilateral PNX (Figure 4B). The protein levels of VEGFR2 and Tie2 in the 24M old mouse lungs after PNX were also restored by LRP5 overexpression (Figure 4C). LRP5 overexpression did not have significant effects on lung growth and vascular and alveolar morphogenesis in 2M old post-PNX mouse lungs (Figure 4A, B). We also investigated the endothelial and alveolar epithelial cell proliferation in 2M vs. 24M old mice after PNX and the effects of LRP5 overexpression using FACS analysis of BrdU^+^ cells (Supplementary Figure S1E). 1.4% and 0.3% of CD31^+^, VE-cadherin^+^, CD45^-^ endothelial cell populations were BrdU-positive in 2M and 24M old sham-operated mouse lungs, respectively. BrdU-positive CD31^+^, VE-cadherin^+^, CD45^-^ endothelial cell populations increased to 11.5% after PNX in 2M old mouse lungs, while increased only to 0.6% in 24M old post-PNX mouse lungs. LRP5 overexpression significantly increased BrdU-positive CD31^+^, VE-cadherin^+^, CD45^-^ endothelial cell populations in the 24M old mouse lungs, while there was no significant effect in the 2M old mouse lungs. Similar trends were observed in EpCAM^+^ alveolar epithelial cell populations. 1% of EpCAM^+^ cells were BrdU-positive in both 2M and 24M old sham-operated mouse lungs. BrdU-positive EpCAM^+^ cell populations increased to 2.7% in 2M old mouse lungs after PNX, while increased only to 1.2% in post-PNX 24M old mouse lungs. LRP5 increased BrdU-positive EpCAM^+^ cell proliferations in the 24M old mouse lungs after PNX. These findings suggest that LRP5 overexpression increases angiogenic factor expression and stimulates compensatory lung growth and vascular and alveolar morphogenesis in the 24M old mouse lungs after PNX.

## DISCUSSION

In this report, we have demonstrated that vascular and alveolar structures are disrupted in the 24M old mouse lungs. The expression of LRP5 and major angiogenic factor receptors, VEGFR2 and Tie2, was lower in ECs isolated from 24M old mouse lungs compared to those from 2M old mouse lungs. Vascular and alveolar formation was suppressed in the fibrin gel implanted on the 24M old mouse lungs and compensatory lung growth after PNX was inhibited in 24M old mice, while these effects were restored by overexpression of LRP5. These results suggest that age-dependent decline in LRP5 expression decreases the expression of angiogenic factor receptors and impairs angiogenesis and alveolar morphogenesis in the aged mouse lungs. Modulation of LRP5 expression may be one of the promising strategies for age-related lung diseases and delaying the aging processes in the lungs.

LRP5 controls various angiogenic pathways (e.g., angiopoietins (Angs)-Tie2 [15,17], VEGF-VEGFR2 [27,28], neuropilin (NRP2) [29]), and stimulates lung vascular development in neonatal mice [14–17,49] and compensatory lung growth after PNX in adult mice [33]. Given that the cooperative action of multiple angiogenic pathways are required for optimal generation and maintenance of physiological and functional blood vessels [50–55] and subsequent organ morphogenesis, manipulation of the expression of LRP5 could be a promising strategy to reverse age-related decline in lung vascular and alveolar formation. LRP5 overexpression may simultaneously stimulate the expression of antagonistic genes, which may eliminate the desired angiogenic phenotype and functions. However, such antagonistic pathways may also be necessary for the well-organized spatiotemporal control of angiogenesis. Consistent with others’ reports [44,45], LRP5 is expressed not only in ECs but in other cell types as well (Supplementary Figure S1C), which influences lung vascular and alveolar morphogenesis. We have found that LRP5 DNA systemic injection restores vascular and alveolar morphogenesis in the post-PNX aged lungs. LRP5 DNA systemic injection increased LRP5 expression in multiple cell types (ECs, immune cells) and may restore vascular and alveolar morphogenesis and compensatory lung growth through signaling in the multiple cell types in the aged lung. Furthermore, LRP5 DNA systemic injection may increase LRP5 expression in other organs, which indirectly affects lung vascular and alveolar morphogenesis. Further investigation using conditional Lrp5 transgenic mice, in which LRP5 expression is manipulated in specific cell types, would elucidate the mechanism by which aging impairs angiogenesis and alveolar morphogenesis. Continuous activation of LRP5 may have additive toxicity resulting from the control of multiple genes and promote tissue fibrosis [56–58], various types of cancer and tumor metastasis [59,60], valve degeneration and calcification [61,62], and osteoarthritis [63,64]. EC specific gene manipulation during specific time frame will maximize the regenerative ability of LRP5 and minimize the potential toxicity.

Overexpression of LRP5 only partially restored angiogenesis and alveolar morphogenesis in the aged lung (Figs. 3, 4). This may be because aged senescent cells secrete a number of cytokines, growth factors, and proteases, which results in diverse inhibitory effects on angiogenesis and lung alveolar regeneration in aged lungs [65,66]. Other signaling pathways associating with LRP5 (e.g., LRP6, TGF-β, Twist1) [67,68] may also be altered during the aging processes and contribute to inhibition of vascular and alveolar morphogenesis. For example, the expression of the transcription factor Twist1, which also controls Tie2 [69] and other angiogenic genes (e.g., PDGF [70], VEGFR2 [71]), is regulated by LRP5 and controls cellular senescence [68,72].

To visualize the newly formed vascular and alveolar morphogenesis in the mouse lungs, we implanted fibrin gel on the mouse lung. Vascular structures in the aged lung tissue and in the gel implanted on the aged mouse lung seem to be different; blood vessel formation was inhibited and only small immature vasculatures were formed in the gel implanted on the aged lung (Figure 3), while dilated tortuous blood vessels were accumulated in the alveolar septa in the aged mouse lung (Figure 1). This may be because of the differences in the microenvironment between the gel and the lung tissues or the experimental time course. Alternatively, impairment of neovascularization in the aged lung (as observed in the gel) may disturb homeostasis of aged blood vessels, and consequently accumulate disrupted blood vessels in the aged lung.

We have demonstrated that mechanical forces control vascular morphogenesis and function [17,73–75]. Appropriate physical properties of lung tissue are necessary for physiological postnatal lung development and LRP5 signaling mediates ECM structure-dependent angiogenesis and alveolar morphogenesis in the neonatal mouse lung [17]. Other mechanosensitive transcription factors and co-activators (e.g., TFII-I, GATA2, Twist1, YAP1) also control angiogenesis [43,48,73,76], and contribute to lung diseases (*e.g.,* pulmonary fibrosis, pulmonary hypertension) [42,43,77]. Aged fibroblasts produce more collagen and less elastin, leading to increasing pulmonary stiffness and lowering compliance [78]. Increases in tissue stiffness in the aged lungs may change the LRP5 expression and/or activity, and contribute to the impairment of angiogenesis and alveolar morphogenesis in the aged lung.

In summary, we have demonstrated that LRP5 mediates age-related decline in vascular and alveolar morphogenesis as well as post-PNX compensatory lung growth in the mouse lungs. Modulation of LRP5 would potentially lead to the development of new therapeutic strategies for aging-associated lung diseases.

## MATERIALS AND METHODS

### Materials

Anti-CD31 antibody was from Transduction Laboratories (Lexington, KY). Anti-AQP5, –SPB, -β-catenin antibodies were from Abcam (Cambridge, MA). Anti-β-actin monoclonal antibody was from Sigma (St. Louis, MO). Anti-VEGFR2 and LRP5 antibodies were from Cell Signaling (Danvers, MA). Anti-Tie2 monoclonal antibody was from Upstate (Lake Placid, NY). Anti-Tie2 polyclonal antibody was from Santa Cruze Biotechnology (Dallas, TX). Anti-EpCAM, -CD31, -VE-cadherin, and –CD45 antibodies were from BioLegend (San Diego, CA).

### Mouse lung cell isolation

Mouse lung ECs were isolated from C57BL6 mice of different ages (2M and 24M old) using anti-CD31 conjugated magnetic beads as previously reported [42] and sorted by FACS (CD31^+^, VE-cadherin^+^, CD45^-^). Isolated ECs were validated by FACS for EC markers (CD31^+^, VE-cadherin^+^, CD45^-^) before use. Isolated mouse lung ECs were cultured in EBM2 medium containing 5% FBS and growth factors (VEGF, bFGF and PDGF) [42] and were used between passages 1-2. EpCAM^+^ mouse lung epithelial cells were isolated from C57BL6 mouse lungs using FACS sorting. Mouse lung immune cells were collected from bronchoalveolar lavage (BAL) fluid of C57BL6 mice [74].

### Plasmid construction and gene knockdown

The retroviral pOC-LRP5 plasmid was constructed as reported [15,33]. As a control, plasmid with vector only was used. Generation of retroviral vectors was accomplished as reported [15,33,73]. Viral supernatants were collected starting 48 h after transfection, for four consecutive times every 12 h, pooled, and filtered through a 0.45 μm filter. Viral supernatants were then concentrated 100-fold by ultracentrifugation in a Beckman centrifuge for 1.5 h at 16,500 rpm. Mouse lung ECs were incubated with viral stocks in the presence of 5 μg/ml polybrene (Sigma) and 90-100% infection was achieved 3 days later [15,33,73,76].

### Molecular biological and biochemical methods

Quantitative reverse transcription (qRT)-PCR was performed with the iScript reverse transcription and iTaq SYBR Green qPCR kit (BioRad, Hercules, CA) using the BioRad real time PCR system (BioRad). Cyclophilin controlled for overall cDNA content. The primers used for mouse Lrp5, Tie2, Vegfr2, and cyclophilin were previously described [15,33,73].

### Fibrin gel mouse lung implantation

The *in vivo* animal study was carried out in strict accordance with the recommendations in the Guide for the Care and Use of Laboratory Animals of the National Institutes of Health. The protocol was reviewed and approved by the Animal Care and Use Committee of Medical College of Wisconsin. C57BL6 mice (Jackson Laboratory and NIA/NIH rodent colonies) and Lrp5 KO mice (stock no. 005823; Jackson Laboratory, developed by Deltagen Inc [15,79]) were used for the study. Fibrin gel was fabricated as described [41–43,48]. Briefly, we added thrombin (2.5 U/ml) with angiogenic factors (VEGF and bFGF at 100 ng/ml) to the fibrinogen solution (12.5 mg/ml), mixed well, and incubated drops of the mixture at 37 °C for 30 min until they solidified [41–43,48]. We implanted the gel on the mouse lungs of different ages for 7 days as described previously [15,41–43,48]. To manipulate gene expression in the gel implanted on the lung, we treated mice with pOC-LRP5 mixed with jetPEI *in vivo* transfection reagent (retro-orbital injection, twice/week, Polyplus, New York, NY) [43]. The formation of blood vessels and alveolar epithelial morphogenesis are evaluated by counting the number of blood vessels stained positive for CD31 and the area stained positive for alveolar epithelial cell markers (AQP5, SPB) from five different areas of the gel [15,41,43,48]. Fluorescent images are taken on a Leica TCS SP5 confocal laser scanning microscope and morphometric analysis is performed using ImageJ software as we reported [15,41–43,48].

### Microfil casting system

Vascular structure was characterized using the microfil vascular casting system [48,80]. After heparinization, mice were euthanized and the cardiac apex was cut. Microfil (0.5-1 ml, Flow Tech) was injected into the pulmonary arteries through right ventricle. After solidification of Microfil, the lungs were fixed with 4% paraformaldehyde, dehydrated with ethanol, cleared with methyl salicylate, and imaged. Quantification of vasculatures was performed using the AngioTool and ImageJ software programs (NIH).

### Unilateral PNX

Unilateral PNX was performed as described [33,48]. In brief, mice (C57BL6, 2M and 24M old) were anesthetized with isoflurane, and intubated with a 21-gauge cannula and mechanically ventilated at 120 cycle/min with a tidal volume of 10 ml/kg using a rodent ventilator (MiniVent, Harvard Apparatus, Holliston, MA). After ensuring adequate anesthesia, a 1 cm incision was made through the skin, muscle above the left lung along the intercostal space between the fourth and fifth ribs were cut, and thoracotomy was performed. A small retractor was placed to provide access to the thoracic cavity. The left lung was gently lifted through the incision and a 5-0 silk suture was passed around the hilum and tied. The hilum was then transected distal to the tie. The remaining portions of the hilum and tie were returned back to the thoracic cavity. The mouse was extubated and observed for return of spontaneous respirations. Sham operated mice underwent thoracotomy without PNX. Since the cardiac lobe is routinely evaluated for compensatory lung growth [47], the weight of the cardiac lobe was measured and normalized to BW after the experiments. Histological samples were prepared as previously reported [15,33,42,43,48] and morphological analysis of MLI and alveolar numbers was performed as described [15,33,48]. The proliferation of ECs and alveolar epithelial cells in the mouse lungs after PNX or in combination with LRP5 overexpression was analyzed by measuring the number of BrdU^+^ cells using FACS (BD Biosciences BrdU flow kit).

### Statistical analysis

All phenotypic analysis was performed by masked observers unaware of the identity of experimental groups. Error bars (SEM) and *p* values were determined from the results of three or more independent experiments. The F test (for two samples) or the Levene test (for more than two samples) was performed to confirm that the variances are homogeneous. Student’s t-test was used for statistical significance for two groups. For more than two groups, one-way ANOVA with a post-hoc analysis using the Bonferroni test was conducted.

## Supplementary Material

Supplementary Figure
