# Supplementary material for: LRP5 in age-related changes in vascular and alveolar morphogenesis in the lung
Source: Aging (Albany NY). 2019 Jan 5;11(1):89–103. doi: 10.18632/aging.101722 (PMC6339783; doi:10.18632/aging.101722)
Supplement: Supplementary Figure [file aging-11-101722-s001.pdf]

SUPPLEMENTARY FIGURE

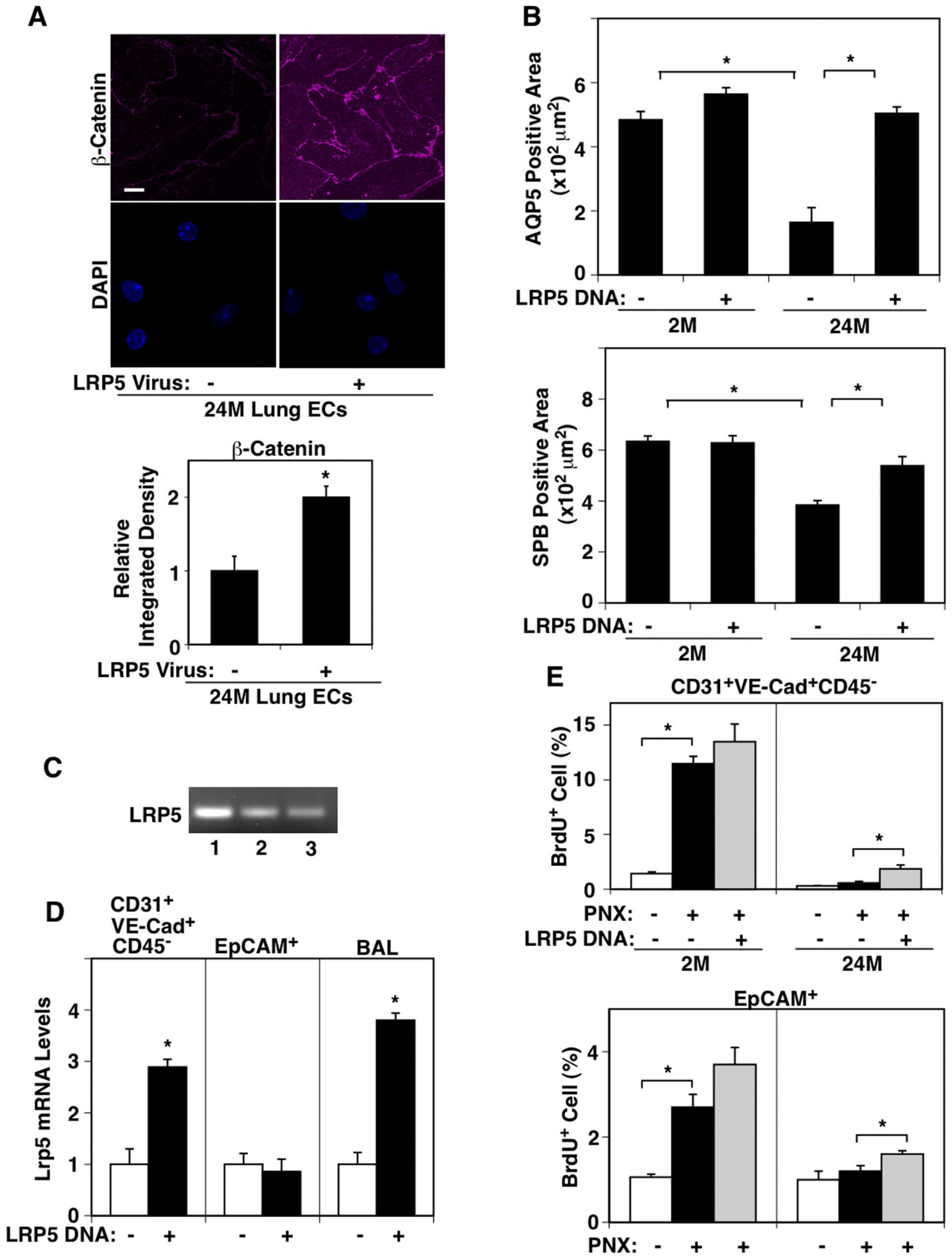

Supplementary Figure S1. LRP5 mediates age-dependent decline in alveolar epithelial morphogenesis in the mouse lungs. (**A**) Immunofluorescence micrographs showing β-catenin expression and distribution in ECs isolated from 24M old mouse lungs treated with lentivirus overexpressing LRP5 (*top*, scale bar, 10 μm). Graph showing the quantification of β-catenin expression in ECs isolated from 24M old mouse lungs treated with lentivirus overexpressing LRP5 (n=4, mean±s.e.m., *p<0.05). (**B**) Graphs showing quantification of AQP5- (*top*) and SPB- (*bottom*) positive epithelial cell area in the gel implanted on the 2M vs. 24M old mouse lungs or in combination with LRP5 overexpression for 7 days (n=7, mean ± s.e.m., *, p<0.05). (**C**) Gel image showing LRP5 mRNA expression in $CD31^+$, VE-cadherin$^+$, $CD45^-$ ECs (*lane 1*), EpCAM$^+$ alveolar epithelial cells (*lane 2*), and immune cells (*lane 3*) isolated from 2M old mouse lungs. (**D**) Graph showing the Lrp5 mRNA levels in $CD31^+$, VE-cadherin$^+$, $CD45^-$ ECs, EpCAM$^+$ alveolar epithelial cells, and immune cells (BAL) isolated from 2M old mouse lungs treated with LRP5 DNA (n=4, *, mean ± s.e.m., *, p<0.05). (**E**) Graph showing the % of BrdU-positive $CD31^+$, VE-cadherin$^+$, $CD45^-$ ECs (*top*) and EpCAM$^+$ alveolar epithelial cells (*bottom*) isolated from 2M vs. 24M old mouse lungs after PNX or in combination with treatment with LRP5 DNA for 7 days (n=5, *, mean ± s.e.m., *, p<0.05).
